# Activation of anaphase-promoting complex by p53 induces a state of dormancy in cancer cells against chemotherapeutic stress

**DOI:** 10.18632/oncotarget.8172

**Published:** 2016-03-18

**Authors:** Yafei Dai, Lujuan Wang, Jingqun Tang, Pengfei Cao, Zhaohui Luo, Jun Sun, Abraha Kiflu, Buqing Sai, Meili Zhang, Fan Wang, Guiyuan Li, Juanjuan Xiang

**Affiliations:** ^1^ Hunan Cancer Hospital and The Affiliated Cancer Hospital of Xiangya School of Medicine, Central South University, Changsha, Hunan, PR China; ^2^ Cancer Research Institute, Key Laboratory of Carcinogenesis and Cancer Invasion of Ministry of Education, Key Laboratory of Carcinogenesis of Ministry of Health, Central South University, Changsha, Hunan, PR China; ^3^ Department of Thoracic Surgery, The Second Xiangya Hospital, Central South University, Changsha, Hunan, PR China; ^4^ Hunan Key Laboratory of Nonresolving Inflammation and Cancer, Changsha, Hunan, PR China; ^5^ Department of Neurology, Xiangya Hospital, Central South University, Changsha, Hunan, PR China

**Keywords:** cancer dormancy, drug resistance, p53, Smad2, DNA repair

## Abstract

Cancer dormancy is a stage in tumor progression in which residual disease remains occult and asymptomatic for a prolonged period. Cancer cell dormancy is the main cause of cancer recurrence and failure of therapy. However, cancer dormancy is poorly characterized and the mechanisms of how cancer cells develop dormancy and relapse remain elusive. In this study, 5- fluorouracil (5-FU) was used to induce cancer cell dormancy. We found that cancer cells escape the cytotoxicity of 5-FU by becoming “dormant”. After exposure to 5-FU, residual non-small cell lung cancer (NSCLC) cells underwent epithelial-mesenchymal transition (EMT), followed by mesenchymal-epithelial transition (MET). These EMT-transformed NSCLC cells were in the state of cell quiescence where cells were not dividing and were arrested in the cell cycle in G0-G1. The dormant cells underwent an EMT showed characteristics of cancer stem cells. P53 is strongly accumulated in response to 5-FU-induced dormant cells through the activation of ubiquitin ligase anaphase-promoting complex (APC/C) and TGF-β/Smad signaling. In contrast to the EMT-transformed cells, MET-transformed cells showed an increased ability to proliferate, suggesting that dormant EMT cells were reactivated in the MET process. During the EMT-MET process, DNA repair including nonhomologous end joining (NHEJ) and homologous recombination (HR) is critical to dormant cell reactivation. Our findings provide a mechanism to unravel cancer cell dormancy and reactivation of the cancer cell population.

## INTRODUCTION

Patients with cancer can develop local or disseminated recurrence after therapy and long periods of latency, which is a major obstacle preventing cure and long-term survival in patients [1]. This latency can be explained by cancer dormancy, a stage in cancer progression in which residual disease is present but remains asymptomatic [1]. Cancer cell dormancy is the main cause of cancer recurrence and failure of therapy [2,3]. Cancer dormancy is characteristic of cell quiescence where cells are not dividing but at arrest in the cell cycle in G0-G1. Disseminated tumour cells (DTC) escaping apoptosis are likely in a dormant cell-cycle arrest stage [4]. Chemotherapy, which uses drugs to destroy cancer cells, is a main type of cancer treatment. Nevertheless, chemoresistance and tumor recurrence after chemotherapy remain a major etiology of the morbidity and mortality in cancer patients [5]. It is assumed that dormant cells are chemotherapy resistant because they are not dividing. Cancer cells evade chemotherapy drugs by becoming “dormant”. However, cancer dormancy is poorly understood, and the mechanisms of how cancer cells develop dormancy and relapse remain elusive. Oncogenic signaling might not always be dominant and stem cell quiescence and stress signaling could overcome oncogenic signals, allowing tumor cell survival in a dormant state [1].

Accumulating evidence suggests that a subpopulation of cancer cells exhibit stem-like properties [6]. It is well recognized that these cancer stem cells (CSCs) are related to cancer recurrence, metastasis and drug resistance. There appears to be a causal relationship between CSCs and the epithelial-to-mesenchymal transition (EMT) in solid tumors [7–10]. Epithelial-mesenchymal transition (EMT), and its reverse process mesenchymal-epithelial transition (MET), refer to the transition in cells between an epithelial phenotype and a mesenchymal phenotype. Emerging empirical evidence suggests that there are plenty of molecular and phenotypic associations between EMT and drug resistance [11–14]. EMT-transformed cells are also linked with decreased proliferation or quiescence [5,15].

In this study, we used the chemotherapeutic drug 5- fluorouracil (5-FU), which is an S phase-active chemotherapeutic agent, with no activity when cells are in G0 or G1 phase, to induce cancer cell dormancy. We found that cancer cells that escape cytotoxicity of 5-FU by becoming “dormant” displayed EMT followed by MET. The dormant EMT cells showed characteristics of cancer stem cells. P53 was strongly accumulated in response to DNA damage and caused cancer cell arrested in G0-G1 through the activation of ubiquitin ligase anaphase-promoting complex. During the EMT-MET process, DNA repair, including nonhomologous end joining (NHEJ) and homologous recombination (HR), is critical to dormant cell reactivation. Our findings provide a mechanism to unravel cancer cell dormancy and regain tumorigenicity. Our study also suggests therapies that prevent relapse by either sustaining tumor dormancy by inhibiting DNA repair or by selectively enhancing chemotherapy sensitivity by inhibiting APC/C activity.

## RESULTS

### Cancer cells escaping cytotoxicity of 5-FU undergo EMT followed by MET

**Figure 1:**
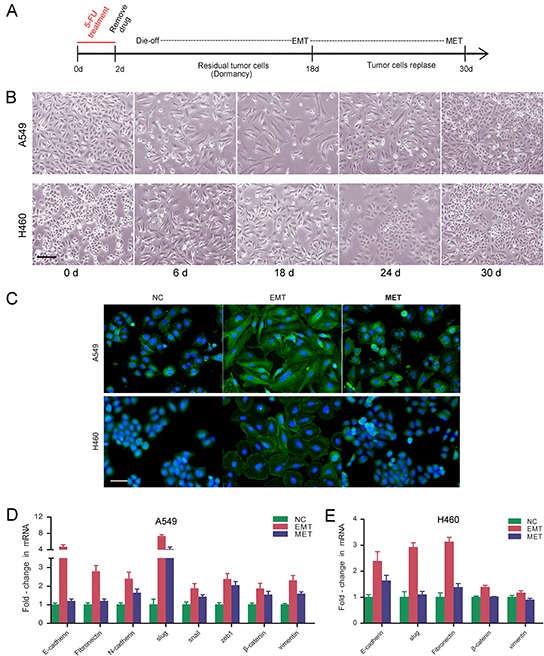
Cancer cells escaping cytotoxicity of 5-FU undergo EMT followed by MET **A.** NSCLC cells were exposed to anti-cancer drug 5-FU. The treatment protocol was summarized. **B.** Representative photos for A549 and H460 after exposure to 5-FU. Photos were taken on day 0, 6, 18, 24, 30. Magnification: ×100; scale bar = 100 μm. **C.** Cytoskeleton actin stained with FITC-phalloidin in EMT- and MET-transformed NSCLC cells. Nuclei were co-stained with DAPI. Expression of epithelial and mesenchymal marker genes in EMT- and MET-transformed NSCLC cells were shown in **D.** A549; **E.** H460. Real-time PCR were performed to measure the relative expression of epithelial and mesenchymal marker genes. GAPDH served as internal control.

NSCLC cells were exposed to the anti-cancer drug 5-FU. The treatment protocol was summarized in Figure 1A. 48 hrs after treatment, most of the cells were killed, whereas the surviving cells acquired a more fibroblastoid-like cell shape. Eighteen days after the removal of 5-FU, the cells returned to a polygonal epithelial-like state (Figure 1B). We stained the cells with phalloidin-labeled F-actin. F-actin was assembled into actin stress fibers in cells underwent EMT, which appeared to have increased intercellular separation and showed increased formation of pseudopodia. In contrast, cells underwent MET displayed cell-cell junction and little formation of pseudopodia (Figure 1C). We examined the expression of the epithelial marker E-cadherin and mesenchymal markers, such as N-cadherin, vimentin, fibronectin, snail, and slug in these two processes. Enhanced mRNA expression levels for mesenchymal markers were observed in EMT, along with an enhanced mRNA expression level for E-cadherin. In MET, the expression levels of the typical mesenchymal markers were obviously decreased compared with those in EMT (Figure 1D, Supplementary Figure S1).

### APC/C signaling pathways are activated in dormant cells underwent an EMT

**Figure 2:**
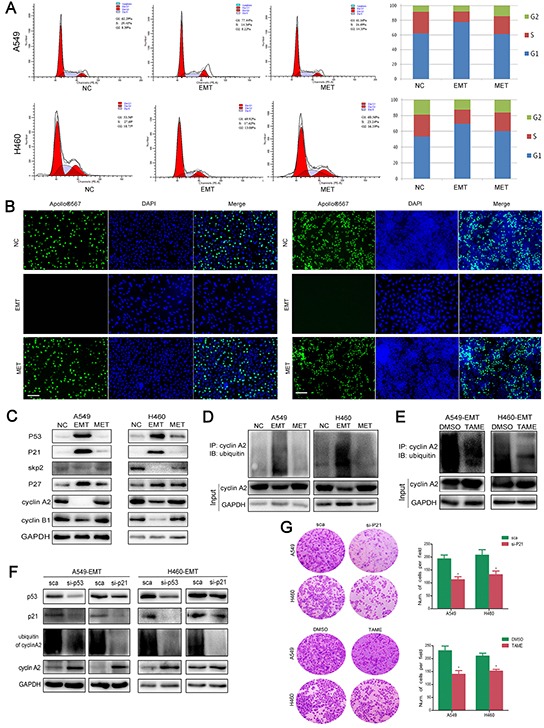
APC/C signaling pathways are activated in dormant EMT cells **A.** Cell cycle analysis was performed in EMT- and MET-transformed cells. Histogram showed the proportion of cells in G0/G1, S and G2/M phases of the cell cycle. **B.** EdU cell proliferation assay was performed to detect the proliferation ability of EMT- and MET-transformed cells. The proliferating cells were labeled with fluorescence labeled-EdU. Magnification, ×40; scale bar = 100 μm. **C.** Expression of cell-cycle related proteins. Total cellular protein was extracted from A549 (NC, EMT, MET), H460 (NC, EMT, MET), then immunoblotted with p53, p21, p27, skp2, cyclin A2, cyclin B1. GAPDH was used as loading control. **D.** Cell lysate from EMT- and MET-transformed NSCLC cells were immunoprecipitated with cyclin A2 antibody and immunoblotted using anti-Ub antibody. GPADH served as internal loading control. **E.** TAME (Tosyl-L-Arginine Methyl Ester) prevent APC-dependent ubiquitination of cyclin A2 in EMT-transformed NSCLC cells. **F.** Knockdown of p53 and p21 prevent ubiquitination of cyclin A2. GPADH serves as internal loading control. **G.** Knockdown of p21 or inhibiting APC/C by TAME sensitized NSCLC cells to 5-FU. Cells were treated with 5-FU. 2 days after treatment, surviving cells were stained with cystal violet. Representive microscopic fields were shown. The blue stained cells were counted and shown in histogram at the right side.

Cell cycle analysis showed that S-phase entry was blocked in NSCLC cells that underwent EMT, and cells were arrested in G0/G1 phase after exposure to 5-FU (Figure 2A). EMT-transformed cells showed decreased proliferation detected using an EdU assay, indicating cells that underwent EMT were in their dormant stage. On the other hand, following EMT transformation, MET-transformed cells showed increased proliferation similar to untreated control cells (Figure 2B), indicating that dormant cells were capable of regaining tumorigenicity during MET.

The cellular response to DNA damage is controlled by the tumor suppressor *p53*, which results in cell-cycle arrest followed by DNA repair. Following treatment with 5-FU, the expression level of *p53* was increased in the residual NSCLC cells that underwent EMT (Figure 2C). To investigate if the cell cycle regulation was mediated by anaphase-promoting complex or cyclosome (APC/C) activation, we performed an ubiquitination assay in which cells underwent sequential EMT and MET. The immunoprecipitation was performed with an antibody recognizing cyclin A2, followed by detection of endogenously ubiquitinated proteins with an anti-ubiquitin antibody. The amount of ubiquitinated cyclin A2 (essential for G1/S and the G2/M transitions) was increased in EMT-transformed NSCLC cells (Figure 2D). Treatment with the APC/C inhibitor TAME led to the accumulation of non-degraded cyclin A2 (Figure 2E). To further investigate if the ubiquitin lagase function is p53-dependent, we electro-transfected p53 siRNA and p21 siRNA in EMT- and MET-transformed cells. Knockdown of p53 and p21 led to an increase of cyclin A2 and decrease of ubiquitinated cyclin A2 (Figure 2F). We then verified that the APC/C substrates SKP2, cyclin A2, cyclin D1 were degraded in EMT-transformed cells. The changes in p27, p21, and p53 levels were inversely related to the changes in Skp2 levels (Figure 2C). Knockdown of p21 or inhibition of APC/C by TAME sensitized NSCLC cells to 5-FU (Figure 2G). However, knockdown of p53 did not enhance the sensitivity of 5-FU, indicating the dual roles of p53 in apoptosis and DNA repair (data not shown). Consequently, we demonstrated that 5-FU induced cancer cell dormancy through the activation of APC/C which is dependent on p53.

### 5-FU-induced dormant EMT-transformed cells display characteristics of CSCs

**Figure 3:**
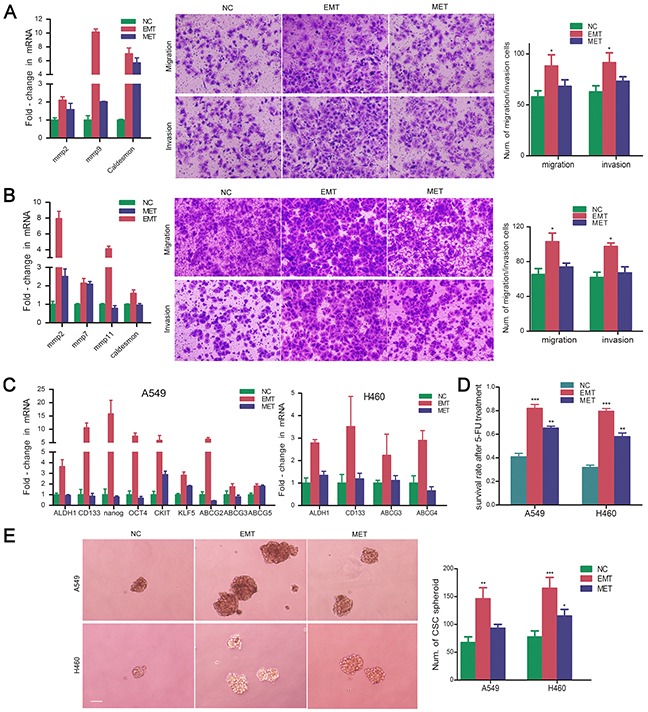
5-FU-induced dormant EMT-transformed cells display characteristics of CSCs Real-time PCR were performed to evaluate the expression of invasion and migration-related genes MMP family and F-actin related gene Caldesmon. Cell migration and invasion was determined using transwell chambers. Cell invasion was assayed in transwell coated with Matrigel. Cells were suspended in serum-free medium and seeded on 24-well transwell plates. Migrated or invasive cells through pores were fixed with ethonal and stained with crystal violet. Expression of MMP family and caldesmon were shown in EMT- and MET-transformed NSCLC cells. The representative images of migrating or invading cells were pictured. Six random microscopic fields were counted. The histograms represented the mean±S.D. **A.** EMT-transformed A549 cells; **B.** EMT-transformed H460 cells; *P<.05, when compared with NC. **C.** Expression of CSC related genes in EMT- and MET-transformed NSCLC cells; **D.** Survival rate of EMT- and MET-transformed NSCLC cells after exposure to 5-FU. **P<.01; *** P<.001, when compared with NC. **E.** Spheroid formation of EMT- and MET-transformed NSCLC cells. Representative images of sphere-forming NSCLC cells were shown. Magnification, ×100; scale bar = 150 μm. The number of spheres was counted. The histograms represented the mean±S.D. (*P<.05; **P<.01; *** P<.001, when compared with NC).

The acquisition of an EMT phenotype is associated with tumor aggressiveness and metastasis. Chemotherapy-induced EMT-transformed NSCLC cells showed enhanced migration and invasion compared with untreated control and MET cells, with higher expression of metastasis-related molecules MMP2, MMP9, and caldesmon (Figure 3A, 3B). These EMT-transformed NSCLC cells exhibited increased expression of CSC marker genes including *CD133, ALDH1, nanog, oct4, c-kit, KLF5, ABCG2, ABCG5*, and others (Figure 3C). EMT-transformed cells showed more resistance to 5-FU than untreated control and MET-transformed cells. The sphere formation ability of EMT-transformed cells was significantly enhanced compared with that of untreated control and MET-transformed cells (Figure 3D). Overall, it was demonstrated that the cells that underwent EMT after 5-FU treatment displayed CSC characteristics.

### 5-FU induced dormant EMT-transformed cells are mediated by TGF-β/smad-slug pathway

**Figure 4:**
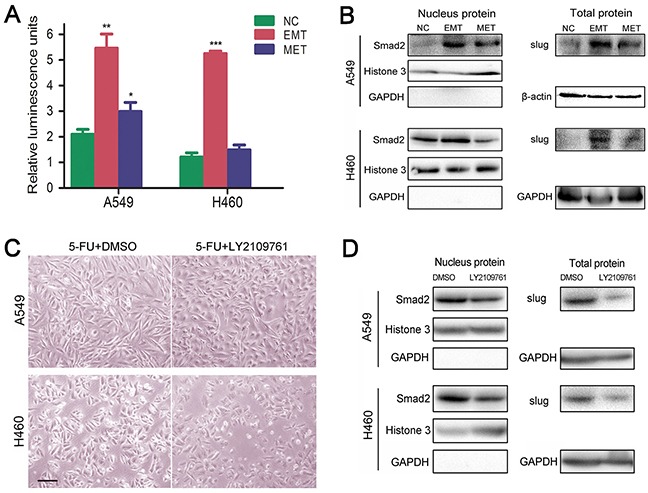
5-FU induced dormant EMT-transformed cells are mediated by TGF-β/smad-slug pathway **A.** Relative luminescence value in NC, EMT, MET stage of A549 and H460, which were transfected with SMAD luciferase reporter plasmid. (*P<.05; **P<.01; ***P<.001, when compared with NC). **B.** Up-regulated expression of slug and nuclear translocation of Smad2 in EMT- and MET-transformed NSCLC cells. Western blot analysis was performed to measure the expression of slug and location of smad2. Histone 3, GAPDH or β-actin were used as loading control for nuclear and cytoplasm proteins respectively. **C.** TGF-β/smad pathway inhibitor LY2109761 suppressed 5-FU-induced EMT. Magnification, ×40; scale bar = 100 μm. **D.** LY2109761 inhibited nucleus entrance of smad2 and slug's expression in 5-FU-induced EMT.

The TGF-β/smad pathway is a canonical pathway in the regulation of EMT; meanwhile, blockade of the pathway was shown to stimulate MET [16]. To identify whether the pathway is involved in 5-FU induced EMT-MET process, we co-transfected SMAD luciferase reporter plasmid and the pRL-TK vector (10:1) into untreated control, EMT- and MET-transformed NSCLC cells. As a result, NSCLC cells underwent EMT showed increased luciferase activity (Figure 4A). In EMT- and MET-transformed cells, increased expressions of Smad2 and slug were detected. Smad2 was distributed predominantly in the nucleus of EMT-transformed cells. There was a corresponding decline in both smad2′s nuclear translocation and slug's expression in cells underwent MET (Figure 4B). LY2109761, the TGF-β/smad pathway inhibitor, can reverse 5-FU-induced EMT and inhibit the expression of slug and nuclear translocation of Smad2 in NSCLC cells underwent EMT (Figure 4C, 4D). Considering the above, we can conclude that 5-FU induced the EMT-MET process through TGF-β/smad-slug signaling.

### DNA repair is activated due to genotoxicity caused by 5-FU during the EMT-MET program

**Figure 5:**
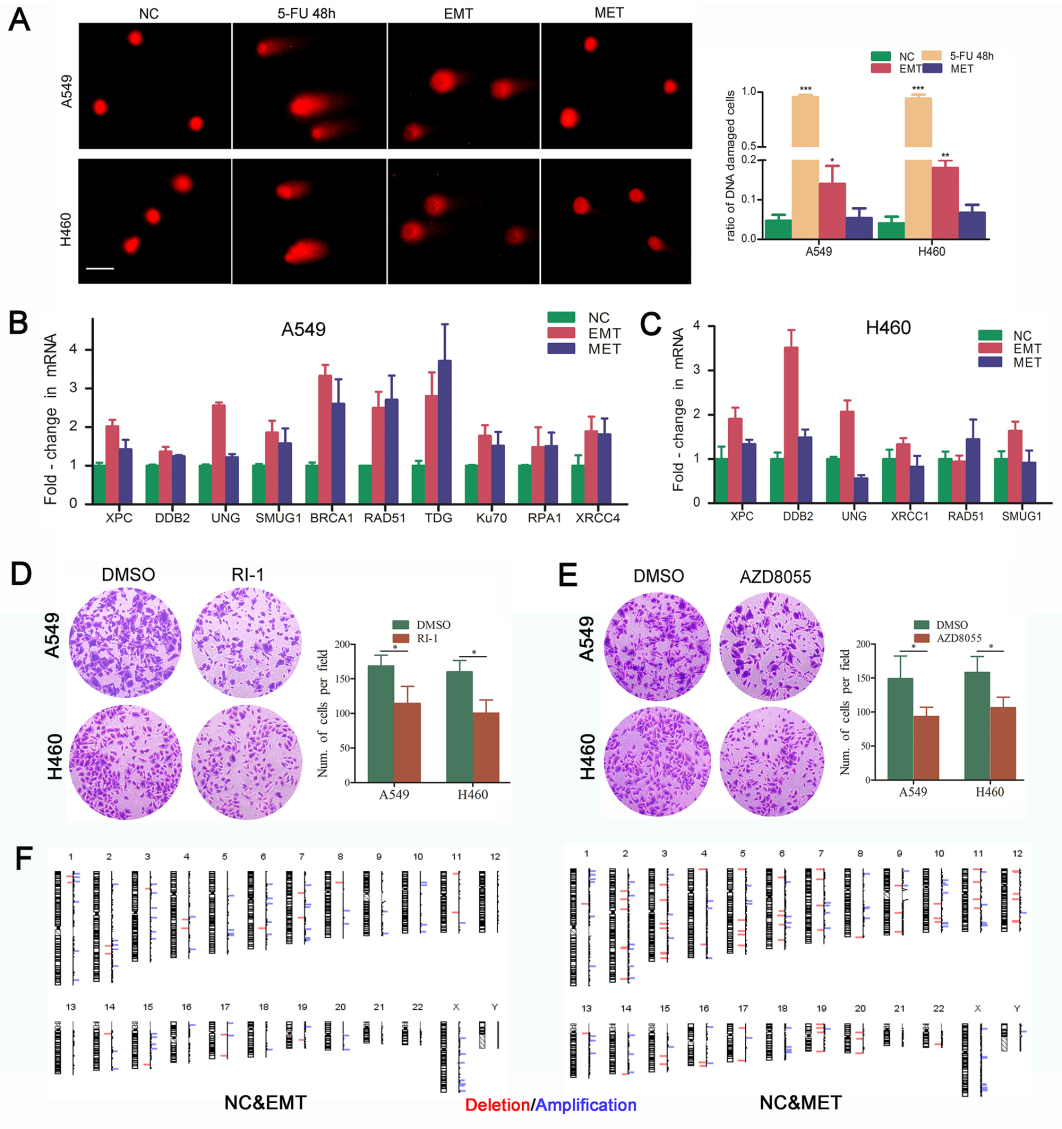
DNA repair is activated due to genotoxicity caused by 5-FU during the EMT-MET program **A.** DNA damage induced by 5-FU treatment. Comet assay was applied to detect the DNA damages in NSCLC cells after exposure to 5-FU. Single cells were electrophoresed in agarose gel on a coverslip and stained with propidium iodide as described in the Methods section. Labeled DNA was visualized under fluorescence microscope. Cells with damaged DNA displayed comet tails. Magnification, ×100; scale bar = 50 μm. Quantitative analysis of cells with damaged DNA in each group was presented. (*P<.05; **P<.01; *** P<.001, when compared with NC). Expression of DNA repair-related genes in EMT- and MET-transformed NSCLC cells were shown in **B.** A549; **C.** H460. Real-time PCR was used to measure the mRNA expression of DNA repair-related markers. GAPDH served as internal control. **D.** Inhibiting RAD51 by RI-1 or AZD8055 sensitized NSCLC cells to 5-FU. **E.** Inhibiting DNA-PK by AZD8055 sensitized NSCLC cells to 5-FU. Cells were treated with 5-FU. 2 days after treatment, surviving cells were stained with cystal violet. Representive microscopic fields were shown. Magnification,×100; The blue stained cells were counted and shown in histogram at the right side. The histograms represented the mean±S.D. (*P<.05; when compared with NC). **F.** Graphic presentation of all chromosomal changes. EMT and MET cells were analysed by comparative genomic hybridization array (Array-CGH). The regions of DNA gain (*blue*) and loss (*red*) were shown.

G1 phase arrest is always accompanied by DNA repair. We further investigated the efficiency of DNA repair during cell cycle in 5-FU induced EMT-transformed cells. A COMET assay showed long comet tails after treatment with 5-FU, and the comet tails were shorter in the cells underwent EMT, which is indicative of DNA repair during EMT. There were no substantial differences in the comet tails between MET-transformed cells and unexposed control cells, indicating that DNA repair occurs in both EMT and MET processes (Figure 5A, 5B). The expressions of the NER, BER, HR and NHEJ pathway DNA repair-related molecules, including *XPC, DDB2, BRCA1, RAD51, KU70,* and others were increased in cells underwent EMT. NER and BER pathway DNA repair-related molecules including *XPC, DDB2, UNG, SMUG1,* and *XRCC1* were decreased in MET-transformed cells compared with EMT-transformed cells. However, the activation of imprecise repair HR and NHEJ pathways was maintained in MET-transformed cells, which is consistent with the regained ability to proliferate in MET (Figure 5C). RI-1 and AZD8055, which are RAD51 inhibitor and DNA-PK inhibitor respectively, could sensitize NSCLC cells to 5-FU (Figure 5D, 5E). In an Array-CGH assay, the evaluation of DNA copy number changes was performed by comparing a DNA test isolated from A549 cells underwent EMT or MET against a normal reference DNA of control A549 cells. A graphical presentation of the regions of gain (blue) and loss (red) was shown in Figure 5F. These abnormalities in cells underwent EMT included gains in chromosome 18,19 and chromosome X. Losses in chromosome 17 and 19 were shown. Compared to EMT, cells underwent MET showed more copy number abnomalities (Figure 5F).

## DISCUSSION

**Figure 6:**
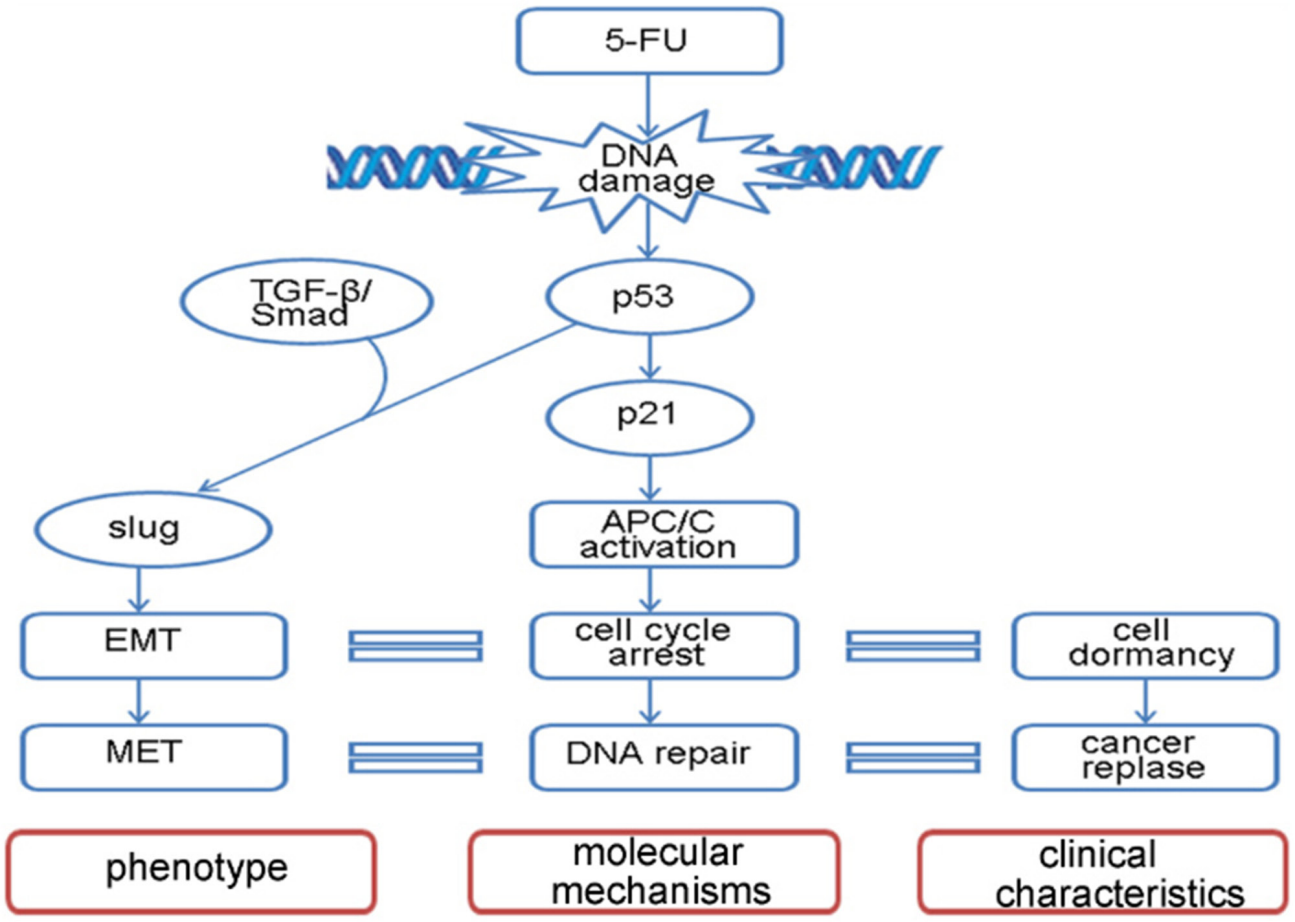
Model diagram of 5-FU-induced EMT-MET program A depiction of the signaling transduction pathway in EMT-MET process stimulated by 5-FU.

In this study, we found that dormant cancer cells induced by 5-FU underwent EMT and MET, which is driven by the TGF-β signaling pathway. In response to DNA damage, p53 activated APC/C and induced cancer cell cycle arrest in G0-G1. The dormant cancer cells underwent EMT displayed characteristics of CSC. DNA repair occurred during cell dormancy, and eventually the cells continued to multiply, resulting in the reappearance of the cancer (Figure 6).

Dormant cancer cells are believed to be involved in chemotherapy resistance, cancer recurrence and metastases [17,18]. 5-FU and other chemotherapeutic drugs were used to enrich the dormant cancer cells [19,20]. Dormancy is a state in which cancer cells aggressively escape damage-induced apoptosis and repair cytotoxic lesions, rather than a passive response to pharmaceutical therapy. Potential therapeutic approaches for dormancy are now drawing more attention from basic and clinical researchers [21]. Several mechanisms can explain cancer dormancy, including impaired angiogenic capacity, immunosurveillance and the disruption of crosstalk between growth factor and adhesion signaling [1, 22]. Inhibition of ERK signaling was observed in dormant cancer cells and led to cellular tumor dormancy through G0-G1 arrest [1, 23]. Stem cell quiescence and stress signaling might overcome oncogenic signals, allowing tumor cell survival in a dormant state [1,17,18]. The TGF-β/smad signaling pathway plays a critical role in regulating the fate of stem cells [24]. Smad2 is one of the key components downstream of the TGF-β signaling pathway. It was well documented that Smad2 promotes CSC self-renewal and maintenance [8, 25–27]. The TGF-β/smad pathway is also the key activator in EMT. Our experimental data showed that the TGF-β/smad pathway was directly involved in 5-FU induced EMT-MET. During the EMT-MET process, Smad2 was shown to be translocated to the cell nucleus. Slug is an important EMT transcription factor and could be directly upregulated by the TGF-β/smad pathway [28]. Similar results were found for Slug expression. Inhibition of the TGF-β/smad pathway with LY2109761 prevented 5-FU-induced EMT and translocation of Smad2 to the nucleus. This demonstrated that activation of TGF-β/smad-slug pathway induce cancer dormancy.

5-FU is a fluoropyrimidine anticancer drug that disrupts cellular metabolism by inhibiting synthesis of purines and pyrimidines, thus disrupting DNA synthesis and RNA translation in target cells [29]. P53 is the core sensor in response to DNA damage. Under multiple cellular stress conditions, p53 functions to block cell cycle progression transiently unless proper DNA repair occurs [30–33]. APC/C is a type of multifunctional ubiquitin-protein ligase that targets various substrates for proteolysis inside and outside of the cell cycle [34]. In the regulation of the DNA damage checkpoint response, APC/C is activated and promotes a broad range of substrates’ proteolysis, such as cyclin A, skp2, cyclin B, securing, etc. [35]. Recent studies revealed that APC/C can be activated in a p53 and p21-dependent DNA damage response [36,37]. p53 signaling is also involved in the maintenance of mouse ESC self-renewal by upregulating nanog [38]. In this study, after exposure to DNA damage drugs, the residual cancer cells underwent a process of cell cycle arrest in the p53-p21-APC/C dependent pathway. Activation of the p53-p21-APC/C pathway contributed to cell cycle arrest through ubiquitin-dependent degradation of cyclin A2 and cyclin B1. In addition, the TGF-β/smad cascade could activate the APC/C complex, mediating Skp2 degradation and cell cycle arrest [39], suggesting that the TGF-β/smad pathway may interact with p53-p21-APC/C to synergistically regulate cell cycle arrest induced by 5-FU. Accordingly, we assume that inhibiting the p53-p21-APC/C pathway may enhance chemotherapy efficacy of 5-FU. As proposed, knockdown of p21 or inhibition of APC/C by TAME could obviously increase cancer cells’ chemosensitivity to 5-FU, which is consistent with studies demonstrating that TAME could inhibit the mitotic block and increase paclitaxel efficacy [40].

There appears to be a causal relationship between CSCs and EMT in solid tumors [10]. EMT and its reverse process MET represent cells not only with transition of phenotype, but also the alteration of oncogenic characteristics. In our study, EMT induced by 5-FU induce cancer cells with CSC characteristics. However, enhanced mRNA and protein expression levels of E-cadherin were observed in the 5-FU-induced fibroblastoid-shape cells (Figure 1, Supplementary Figure S1). E-cadherin is expressed in most normal epithelial tissues [41]. Although downregulation of E-cadherin is a very typical marker of EMT, loss of E-cadherin expression is not a prerequisite for EMT and cell motility in some specific situations [42–45]. The selective loss of E-cadherin can cause dedifferentiation and invasiveness in human carcinomas, leading E-cadherin to be classified as a tumor suppressor [46]. Other research also found that expression level of E-cadherin was increased in dormant cancer cells [47]. E-cadherin can induce an increase in the level of the cyclin-dependent kinase inhibitor p27(kip1) and a late reduction in cyclin D1 protein [48]. We presume that, like p53, E-cadherin arrests cancer cells in G0-G1 phase and induces cancer cell dormancy. Knocking down the expression of E-cadherin enhanced the sensitivity of cancer cell to 5-FU (Supplementary Figure S1).

How dormant cancer cells are reactivated and regain malignant proliferation remain to be elucidated. In our experiment, we found through the EMT-MET process, residual cancer cells escape the cytotoxicity of 5-FU by becoming dormant and regain malignant proliferation ability by DNA repair. G1 phase arrest is always accompanied by DNA repair. In this respect, we sought to investigate the efficacy of DNA repair after DNA damage. A comet assay showed that 5-FU caused DSBs, and, in the EMT-MET process, the DSBs were repaired. Cells that underwent EMT and MET showed activation of XPC, XRCC1, BRCA1, RAD51, KU70, etc., indicating that dormant cells utilize DNA repair to regain the ability to proliferate. 5-FU-induced DNA damage triggered most DNA repair pathways including nucleotide excision repair (NER), base excision repair (BER), homologous recombination (HR) and nonhomologous end joining (NHEJ). However, in MET, DSB were repaired mainly by NHEJ. DNA DSBs can be repaired by one of two major pathways: non-homologous end-joining (NHEJ) and homologous recombination (HR), depending on whether cells are in G1 or S/G2 phase, respectively [49]. There are canonical and alternative pathways of NHEJ involved in end-joining of the broken DSBs [50]. In contrast to canonical NHEJ, which is considered as a guardian of genomic stability, alternative NHEJ is considered a major source of genomic instability [51,52]. Emerging evidence suggest that defects in a main repair pathway for DSBs and NHEJ lead to upregulation of an alternative or “back-up” repair that can create chromosomal deletions and translocations [49,53]. Our data suggest that the NHEJ pathway that is activated during MET increases the genomic instability of NSCLC cells and allows dormant EMT-transformed cells to regain ability to proliferate.

This study provides an *in vitro* model that uses 5-FU to induce dormant EMT-transformed cells and reactivated MET-transformed cells. It is important to investigate the cellular or serum biomarkers to help detect the dormant status. This study also offers two possibilities : First, to prevent reactivation of dormant cancer cells by inhibiting NHEJ DNA repair, and second, to enhance chemotherapy sensitivity by inhibiting APC/C activity and reducing chemotherapy-resistant dormant cancer cells.

## MATERIALS AND METHODS

### Reagents

5-Fluorouracil (F6627) was purchased from Sigma-Aldrich Sigma-Aldrich, Saint Louis, MO, USA (Saint Louis, MO, USA) Sigma), TAME (APC/C complex inhibitor, S2225). LY2109761(TGF-β/smad inhibitor, S2704). RI-1 (RAD51 inhibitor, S8077) and AZD8055 (DNA-PK inhibitor, S1555) were purchased from Selleck Chemicals (Houston, TX, USA).

### Cell culture

Human NSCLC cell lines A549 and H460 were cultured in RPMI-1640 medium (Hyclone, Life Sciences, USA) supplemented with with penicillin G (100 U/mL), streptomycin (100 mg/mL) and 10% fetal calf serum. Cells were grown at at 37°C in a humidified atmosphere of 5% CO2 and were routinely sub-cultured using 0.25% (w/v) trypsin-EDTA solution. CO2. Cells were treated with 5-FU at 80μM for A549 and 20μM for H460 for 48h.

### Quantitative real-time PCR

Total RNA was isolated from cells using Trizol® reagent (15596-026, Invitrogen, USA). cDNA was synthesized from the total RNA with RevertAid First Strand cDNA Synthesis Kit (K1622, Fermentas, USA). Real-time PCR was performed using the Bio-Rad IQTM5 Multicolor Real-Time PCR detection System (Bio-Rad, USA). The data were analysed using iQ5 software. mRNA PCR quantification used 2ΔΔCt method against the GAPDH for normalization. The data are representative of the means of three experiments. Student's t-test was applied to compare two or more values; P <.05 indicated that there was a significant difference. The qPCR protocol was 95°C for 30 sec and 40 cycles of 95°C for 5 sec and 60°C for 30 sec. A final melting curve analysis (60–95°C) was conducted. The standard curve was produced using a slope of approximately -3.32 (~100% efficiency). RT-PCR primers were listed in Table 1.

**Table 1:** Primer sequence for real-Time PCR

Gene	Primer (Forward)	Primer (Reverse)
fibronectin	ACAAGCATGTCTCTCTGCC	TTTGCATCTTGGTTGGCTGC
slug	AGATCTGCCAGACGCGAACT	GCATGCGCCAGGAATGTTCA
snail	TCAAGATGCACATCCGAAGCC	TTGTGGAGCAGGGACATTCG
zeb1	GCACAACCAAGTGCAGAAGA	GCCTGGTTCAGGAGAAGATG
vimentin	CGGGAGAAATTGCAGGAGGAGA	TCTTGGCAGCCACACTTTCAT
β-catenin	GACGTTGACTTGGATCTGTC	GACGTTGACTTGGATCTGTC
E-cadherin	TGAAGCCCCCATCTTTGTGC	GGCTGTGTACGTGCTGTTCT
mmp2	CATTTGGCGGACTGTGAC	GGGTGCTGGCTGAGTAGAT
mmp7	GGCTTTAAACATGTGGGGCA	GACTGCTACCATCCGTCCAG
mmp9	TCCACCCTTGTGCTCTTCC	GCCACCCGAGTGTAACCAT
mmp11	TCATGATCGACTTCGCCAGG	CAGTGGGTAGCGAAAGGTGT
caldesmon	TGGAGGTGAATGCCCAGAAC	GAAGGCGTTTTTGGCGTCTT
CD133	GCCTTTCTCCTGCCT	GGGGTCATTCACTCAAGG
ALDH1	GTCCTACTCACCGATTTGAA	CTTGTATAATAGTCGCCCCC
nanog	GAACTCTCCAACATCCTGAA	TATTCTTCGGCCAGTTGTTT
OCT4	AGTGAGAGAATTTTGCAGGT	GTGATCCTCTTCTGCTTCAG
CKIT	TGGTATTTTTGTCCAGGAACT	GATTTGCTCTTTGTTGTTACCT
KLF5	CAATAGAAGGAGTAACCCCG	CACTTGTATGGCTTTTCACC
ABCG2	GAACCCAAGGAGATAGGAGA	CTAGACAGACTTCAACCAGG
ABCC3	CCTTCCAGGTAAAGCAAATG	GTGTCAGGGTAGAGTCCAAT
ABCG4	GCCCCCTATTCCTTCAGTCC	TCCCGTGGCCTTCCATTAAC
ABCC5	TTTTCAGGATGGCTGTATTCT	TGGCTTCTTTTCCAGTATGC
BRCA1	TCCCATCTGTCTGGAGTTGA	TGTGAAGGCCCTTTCTTCTG
XRCC4	TGCAAAGAAATCTTGGGACAG	TGCTCCTTTTTCGACGTCTC
Ku70	GCTAGAAGACCTGTTGCGGAA	TGTTGAGCTTCAGCTTTAACCTG
RPA1	CCGTAGTAATGGGACGGATG	GCAGAAGGGGGATACAAACA
RAD51	ATACTGTGGAGGCTGTTGCC	AGTTGCAGTGGTGAAACCCA
SMUG1	GAAAGGAACCGGAAACGGGA	CTCCTCCTCCAGGAAGCTCT
XRCC1	CTCACCAGTGCTCCAGGAAG	TGTCCGTGTTCTCATCCGTG
TDG	GAGCTTGAGTCCAGCCACTG	GCTGTTCATTCACAACTGCCA
XPC	AACCTGCCCAATCTACACCG	AGACCTTTGGCCAGCAACTT
DDB2	CCCTTTGACAGGAGGGCTAC	AACTTCAGCCCAGTGATGCT
UNG	GTCTCCCCGCTCCAGTTTAG	CTCTGGATCCGGTCCAACTG
GAPDH	AACGGATTTGGTCGTATTGG	TTGATTTTGGAGGGATCTCG

### Cytoskeleton staining

After fixation of 4% (w/v) paraformaldehyde for 20min, cell cytoskeleton staining was performed by using Phalloidin-FITC (Sigma-Aldrich, Saint Louis, MO, USA) at the final concentration of 1μg/ml for 1.5 h at 37°C, then nucleus was co-stained with 4′,6-diamidino-2-phenylindole (DAPI) (C1002, Beyotime Biotechnology, Hangzhou, China) for 3 min at room temperature. Representative pictures were photographed with FSX100 (Olympus) in random fields.

### *In vitro* migration and invasion assays

*In vitro* cell migration and invasion assays were performed using transwell chambers (3422, Corning, USA). For invasion assay, before cell seeding, transwell plates(Corning, 356234) were coated with Matrigel (BD) and placed in the cell culture hood for 1 hour at 37°C. 200 ¼l of the cell suspension (1×105 cells per well) was seeded in the inserts and cultured in serum-free medium. Normal growth medium were placed in underneath wells. The cells were then cultured for 16 h (migration) and 24h (invasion). Migrated cells were fixed in 100% methanol and stained with 0.1% crystal violet (w/v). Invasive cells on the lower surface of the membrane were stained by dipping inserts in the staining solution for 20 minutes. Representative pictures were photographed with FSX100 (Olympus) in random fields. Stained cells were counted in six random fields.

### Spheroid formation of cancer stem cell

Cells were trypsined and resuspended, followed by seeding into ultra-Low attachment 6-well plate (3741, Corning, USA) at the concertration of 5×103 cells per well. Cells were cultured in serum-free DMEM/F12 (11320033,Gibco,), supplemented with 20 ng/mL epidermal growth factor (Life, PHG0314), 20 ng/mL basic fibroblast growth factor (Life, 13256029) and B27 (Life, 17504-044). The tumor sphere growth was analyzed under inverted microscope 8 days after of culture. The number of spheres (Φ>150 μm) in each well was counted. The experiment was repeated three times and 3 parallel samples were measured.

### SMAD luciferase reporter assay

SMAD luciferase reporter plasmid (GM-021043, Genomeditech, USA) was used to investigate whether TGFβ/smad signaling pathway was activated in the process of EMT induced by 5-FU. After seeded in 12-well plate overnight, cells were co-transfected with SMAD luciferase reporter plasmid and Renilla luciferase vector pRL-TK vector (Promega) at a ratio of 10:1. pRL-TK was co-transfected to normalize the transfection efficiency in each experiment. Cells were cultured for additional 36 h. Luciferase activity of each group was measured by Dual-Luciferase Reporter Assay System referring to the manufacturer's instructions(Promega, E1910). The firefly luciferase and renilla activities were measured with PAEASIFMTM Detection Platform (Beckman Coulter). The relative luciferase activity was represented by the ratio of firefly luciferase activity to renilla luciferase activity. Experiments were performed in three independent replicates and data were shown as means.

### Western blot assay

The cell lysate used for western blotting was extracted using RIPA lysis buffer (CW2333, Cwbio, Beijing, China) containing protease inhibitors (Roche, Germany). Nucleoprotein was obtained by using Nucleoprotein Protein Extraction Kit (P0027,Beyotime Biotechnology, Hangzhou, China). Proteins were quantified using the BCA Protein Assay Kit (CW0014,Cwbio, Beijing, China). The western blot system was established using a BioRad Bis-Tris Gel system according to the manufacturer's instructions (Bio-Rad, CA, USA). Briefly, Approximately 50 μg of protein were separated on SDS-PAGE and transferred to PVDF membranes (ISEQ00010,Millipore, Danvers, MA, USA). The membranes were blocked by 5% nonfat milk. The membranes were incubated with the primary antibody overnight at 4°C, followed by a brief wash with PBST and incubation with secondary antibody for 1 h at 37°C. An anti-GAPDH antibody control was purchased from Millipore (Billerica, MA, USA) and was used as a loading control. Finally, ECL solution (WBLUC0100,Millipore,USA) was added to cover the blot surface and the signals were captured and the intensity of the bands was quantified using the Bio-Rad ChemiDoc XRS+ system(Bio-Rad, CA, USA). For detecting the ubiquitination of cyclin A2, cells were treated with MG-132 (20μM, 8h) (M8699,Sigma), then total protein was collected using lysis buffer added with N-Ethylmaleimide (E3876, Sigma,USA). Cyclin A2 was immunoprecipitated with cyclin A2 antibody (4°C, 12h), followed with Protein A/G PLUS-Agarose (Santa Cruz, sc-2003) incubation (4°C, 4h). The precipitates were washed with lysis buffer for 3 times, then eluted and boiled in sample loading buffer and subjected to SDS-PAGE and immunoblot analysis. The antibodies were used as follows: p53 (sc-126, Santa Cruz Biotechnology, CA, USA), p27 (sc-528, Santa Cruz Biotechnology, CA, USA), Ub Antibody (sc-9133, Santa Cruz Biotechnology, CA, USA), β-actin (sc-47778, Santa Cruz Biotechnology, CA, USA); skp2 (ab183039,Abcam, USA); p21 (10355-1-AP, Proteintech,), cyclin A2 (18202-1-AP, Proteintech,), cyclin B1 (55004-1-AP, Proteintech), slug (12129-1-AP, Proteintech); smad2 (86F7, Cell Signaling Technology, Danvers, MA); Histone 3 (AH433, Beyotime Biotechnology, China); GAPDH (TA802563, Origene, USA).

### EdU cell proliferation assay

Cell proliferation was detected with Click-iT®EdU Microplate Assay Kit (C10310-3, Ribobio, Beijing, China). Cells were planted in 24 well plate (1×10^5^ cells/well). EdU (50¼M, 300¼L) which is modified by fluorescence was added into wells and incubated in chamber for 3 hours as the protocol indicates. After EdU staining, cells were counterstained with DAPI. EdU-positive cells was imaged randomly by fluorescence microscope FSX100 (Olympas, Japan) and the ratio of positive ones was counted.

### Si-RNA interference

For small interfering RNA (siRNA) experiments, siRNA duplexes were used to reduce p53, p21 and E-cadherin protein expression, respectively (Ribobio, China). Briefly, cells were trypsinized and 1×10^6^ cells were suspended in 100μl Opti-MEM or medium without FBS, then added with 5 μL of 20 μM siRNA duplexes, and electro-transfected by using Super Electroporator NEPA21 (NEPA GENE, Japan). Cells were then transferred to 35-mm tissue culture dishes with 2 mL RPMI1640 medium. The siRNA sequences for knockdown of target genes are shown in Table 2.

**Table 2:** siRNA sequence for knockdown of target gene

Target gene	siRNA sequence (Sense)
Si-p53	5′ GAAAUUUGCGUGUGGAGUAdTdT 3′
Si-p21	5′ GUGGACAGCGAGCAGCUGAdTdT 3′
Si-E-cadherin	5′ GCAGAAUUGCUCACAUUUCdTdT 3′

### Cell-cycle analysis

Cells (2×10^6^/mL) were fixed with 70% cold ethanol overnight, then incubated in ice-cold PBS containing 50 mg RNase A (Takara, Japan) at 37°C for 1 hour. Cells were treated with propidium iodide at the final concentration of 5μg/ml (ST511, Beyotime Biotechnology, Hangzhou, China), and incubated for 20 minutes at room temperature. Then, cells were subjected to flow cytometry for analysis.

### Cell count assay

After treatment, cells were washed with PBS and fixed with 4% (w/v) paraformaldehyde for 20min. then, cells were stained with 1% (w/v) crystal violet dye (C0121, Beyotime Biotechnology, Hangzhou, China). The number of cells was quantified by counting the cells in six random fields under a lighted microscope.

### Alkaline comet assay

The alkaline comet assay was done according to the procedure of alkaline Comet assay kit (GMS10074.3, Genmed Scientifics, USA) with minor modification. Briefly, 3× 105 cells were pelleted and resuspended in 1mL PBS. 10 ¼L sample of resuspended cells were then mixed with an equal volume of prewarmed low–melting point agarose. The agarose-cell mixture was placed on fully frosted (one side) slide precoated with agarose and spread it gently with a coverslip. After 10 min at 4°C, the slides were immersed in precooled (4°C) lysis solution for 80 min in a dark chamber. After soaking with electrophoresis buffer for 30 min, the slides were subjected to electrophoresis (20V, 140mA) for 20 min. Finally, the cells were stained with 10μg/ml propidium iodide (Beyotime Biotechnology, ST511), and individual cells were viewed using an Olympus FSX100 fluorescence microscope. DNA damage was quantified in at least 50 randomly selected comets per slide, and DNA damaged cells with comet's tail were canculated and statistically analysed.

### Array CGH

The copy number changes of DNA interval among experimental groups were accessed by Agilent SurePrint G3 Human Catalog 2x400K CGH Microarrays (Agilent, CA, USA). Genomic DNA of control A549, EMT and MET-transformed A549 were extracted with Universal Genomic DNA Kit (CW2298S, Cwbio, Beijing, China). The sample purification and quality assessment were performed according to the manufacturer's instructions. Experiments were performed on CapitalBio Microarray Platforms (CapitalBio, Beijing, China). Images analyses were carried out with Feature Extraction(Agilent, Santa Clara, CA, USA). The results were visualised with Agilent CytoGenomics.

### Statistical analysis

Data are presented as the mean ± S.D. from at least three separate experiments. Statistical analyses were performed using GraphPad Prism 5 (GraphPad Software, Inc., CA, USA). Multiple group comparisons were performed using ANOVA with a post hoc test for the subsequent individual group comparisons. A p value of less than 0.05 was considered to be significant.

## SUPPLEMENTARY FIGURES


