# Supplementary material for: Activation of anaphase-promoting complex by p53 induces a state of dormancy in cancer cells against chemotherapeutic stress
Source: Oncotarget. 2016 Mar 18;7(18):25478–92. doi: 10.18632/oncotarget.8172 (PMC5041919; doi:10.18632/oncotarget.8172)
Supplement: Supplementary file 1 [file oncotarget-07-25478-s001.pdf]

SUPPLEMENTARY FIGURE

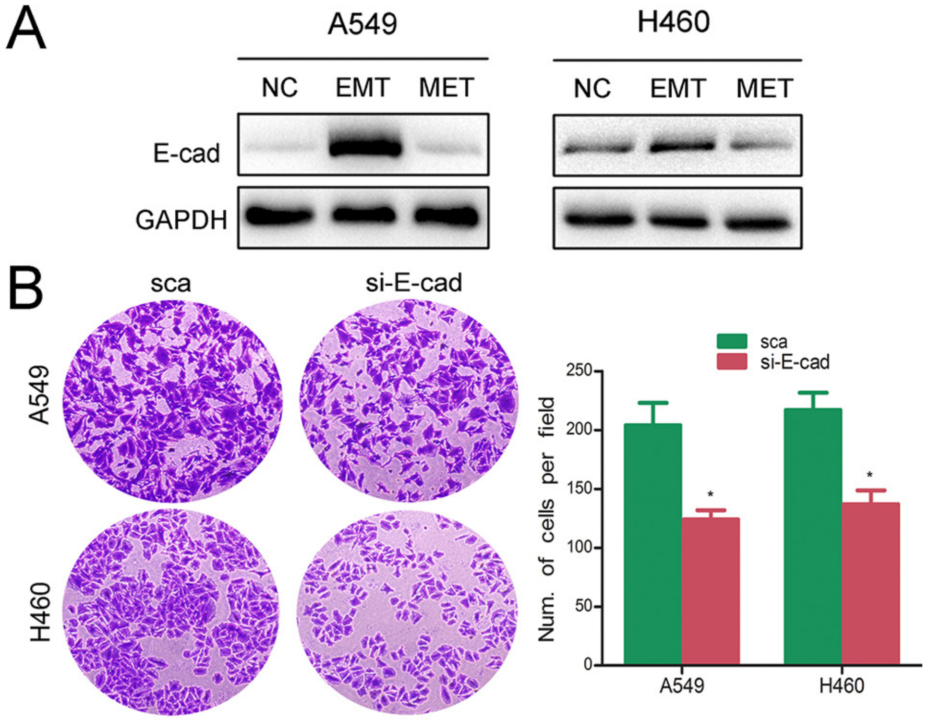

Supplementary Figure S1: Enhanced expression level of E-cadherin in EMT-transformed NSCLC cells. A. Up-regulated expression of E-cadherin in EMT-transformed NSCLC cells. Western blot analysis was performed to measure the expression of E-cadherin. GAPDH was used as loading control. **B.** Knockdown of E-cadherin sensitized NSCLC cells to 5-FU. Cells were treated with 5-FU. 2 days after treatment, surviving cells were stained with cystal violet. Representive microscopic fields were shown. Magnification, ×100; The blue stained cells were counted and shown in histogram at the right side. The histograms represented the mean±S.D. (*P<0.05; when compared with NC).
